# Functional Brain Plasticity Associated with ACL Injury: A Scoping Review of Current Evidence

**DOI:** 10.1155/2019/3480512

**Published:** 2019-12-27

**Authors:** T. Neto, T. Sayer, D. Theisen, A. Mierau

**Affiliations:** ^1^LUNEX International University of Health, Exercise and Sports, Differdange, Luxembourg; ^2^Department of Physiotherapy, The University of Melbourne, Victoria, Australia; ^3^ALAN-Maladies Rares Luxembourg, Luxembourg

## Abstract

Anterior cruciate ligament (ACL) injury is a common problem with consequences ranging from chronic joint instability to early development of osteoarthritis. Recent studies suggest that changes in brain activity (i.e., functional neuroplasticity) may be related to ACL injury. The purpose of this article is to summarize the available evidence of functional brain plasticity after an ACL injury. A scoping review was conducted following the guidelines of the Joanna Briggs Institute and the Preferred Reporting Items for Systematic Reviews and Meta-Analyses. The terms “brain,” “activity,” “neuroplasticity,” “ACL,” “injury,” and “reconstruction” were used in an electronic search of articles in PubMed, PEDro, CINAHL, and SPORTDiscus databases. Eligible studies included the following criteria: (a) population with ACL injury, (b) a measure of brain activity, and (c) a comparison to the ACL-injured limb (contralateral leg or healthy controls). The search yielded 184 articles from which 24 were included in this review. The effect size of differences in brain activity ranged from small (0.05, ACL-injured *vs.* noninjured limbs) to large (4.07, ACL-injured *vs.* healthy control). Moreover, heterogeneity was observed in the methods used to measure brain activity and in the characteristics of the participants included. In conclusion, the evidence summarized in this scoping review supports the notion of functional neuroplastic changes in people with ACL injury. The techniques used to measure brain activity and the presence of possible confounders, as identified and reported in this review, should be considered in future research to increase the level of evidence for functional neuroplasticity following ACL injury.

## 1. Introduction

An anterior cruciate ligament (ACL) rupture is a traumatic knee injury typically affecting young and active people [1–3]. Medical management of a torn ACL involves either conservative treatment or reconstructive surgery, with additional exercise and education strategies to optimise rehabilitation and return to sport [4]. While conservative and surgical management provides positive outcomes at midterm (i.e., 5 years) in young, active adults [5], a high rate of ACL reinjury (≈29.5%) two years following return to sport has been reported [6]. Furthermore, evidence of early onset tibiofemoral and/or patellofemoral osteoarthritis 5-10 years following injury is concerning [7, 8]. This highlights the need for further improvement of current rehabilitation pathways that reduce the risk of second-time ACL injury and/or symptomatic osteoarthritis. Furthermore, primary prevention approaches have received increased focus recently with dedicated conditioning programs, conducted in highly controlled settings, suggesting important reductions in a primary ACL injury risk of ≈50% [9].

Optimising neuromuscular function is considered a key aspect in both prevention [9, 10] and rehabilitation [11]. Most studies have approached this issue from a biomechanical perspective, focusing on kinetics, kinematics, and electromyographic (EMG) activity [12–16]. These studies have substantially advanced our understanding of the functional deficits associated with ACL injury. However, given the established evidence for the role of the brain in sensorimotor control and learning [17–19], it is paramount to identify and evaluate changes in brain function associated with ACL injury.

Recent publications support the notion of functional neuroplasticity in the brain of people with ACL injury [20–22] based on different patterns of brain activity, predominantly in the sensory and the motor areas of the cortex [23]. The disrupted sensory input following ACL injury, together with nociceptor activity related to the inflammatory process, is likely to contribute to changes in the somatosensory feedback [24, 25]. As a consequence, studies have shown reduced maximal voluntary contraction of the quadriceps due to ineffective gamma loop function [26, 27] and increased flexor withdrawal response [28]. Moreover, reconstruction of the ACL does not seem to influence the neuroplastic changes, as patients with ACL reconstruction continue to present changes in neural activity (e.g., increased frontal cortex activity) [29–31]. There is also little information about the time course of brain activity changes following an ACL injury since most of the studies are of cross-sectional design. Moreover, prospective studies, which have the potential to provide valuable information of neuroplastic changes across time, are still scarce [32, 33]. It is therefore not clear whether functional neuroplasticity is a cause or a consequence of ACL injury. In addition, there is not a clear link between the observed differences in brain activity, knee function, and return to sports in people with ACL injury. Studies have shown that athletes who have successfully returned to sports after an ACL injury continued to show changes in brain activity [29, 34]. It seems that particular brain activity adaptations might be relevant for proper knee function and therefore necessary for a successful return to sport.

Another area with serious knowledge gaps concerns the role of brain function as a potential target of rehabilitation following ACL injury. To our knowledge, no study has focused on brain activity changes throughout the rehabilitation process and how these are related to different outcome metrics. Nevertheless, some authors have advanced recommendations for therapies to be implemented with these patients based on observations on brain activity differences. Examples of these interventions range from transcutaneous electrical nerve stimulation (TENS) and cryotherapy [33, 35] to strategies based on external focus of attention and reduced visual feedback [20, 36]. Despite the importance of analysing which intervention could be more suitable according to the differences in brain activity shown by people with ACL injury, it is paramount to have a more comprehensive understanding of the functional neuroplasticity of this population.

The study of neuroplastic changes following ligament injuries is a fairly recent topic with a limited number of studies. Moreover, there is a considerable variety of methods and outcome measures in the study of brain activity which makes it difficult to pool data in a systematic review and meta-analysis format. Thus, a scoping review seems to be the most appropriate method to answer the following research questions: (a) what is the current evidence of differences in brain activity following an ACL injury? (b) What are the potential confounding variables that may influence brain activity following an ACL injury?

## 2. Methods

According to the guidelines from the Joanna Briggs Institute, the most appropriate method to address a research question involving emerging evidence is to conduct a scoping review [37]. We followed the PRISMA guidelines for systematic reviews with the necessary adaptations for a scoping review [38]. No protocol was registered for this scoping review.

### 2.1. Inclusion Criteria

#### 2.1.1. Type of Studies

Following the recommendations of the Joanna Briggs Institute for scoping reviews, different sources of information were considered. Hence, included studies could be from any type of primary research (randomized clinical trials, quasiexperimental studies, cohort studies, case-series studies, cross-sectional studies, case-control studies, and case studies), using human participants with ACL injury and an outcome of brain activity, as measured by electroencephalography (EEG), transcranial magnetic stimulation (TMS), or magnetic resonance imaging (MRI). Letters, commentaries, conference abstracts, and reviews were excluded. Eligible studies needed to include a population with ACL injury, with or without reconstruction; to include a measure of brain activity; and to include any form of comparison to the affected limb (e.g., a healthy control group or the contralateral limb).

#### 2.1.2. Type of Participants

The selected studies had to include participants with unilateral ACL injury, with or without reconstruction, males or females, and from any age category. Participants could present an isolated, primary or recurrent, ACL injury or have concomitant injuries (e.g., menisci or collateral ligament injuries), in addition to the ACL injury. No minimal or maximal time since injury was defined, and all levels of physical activity and knee function were accepted. Studies using people with isolated knee osteoarthritis or using artificially induced knee effusions were excluded.

#### 2.1.3. Types of Outcomes

For the purpose of this review, neuroplasticity was defined as the ability of the CNS to adapt and reorganize following a lesion or environmental change [39]. In order to determine the presence of neuroplastic changes following an ACL injury, the included studies had to use any measure of brain or cortical activity (e.g., EEG, TMS, or MRI). Studies reporting differences only at the spinal level were excluded.

### 2.2. Search Strategy

**Figure 1:**
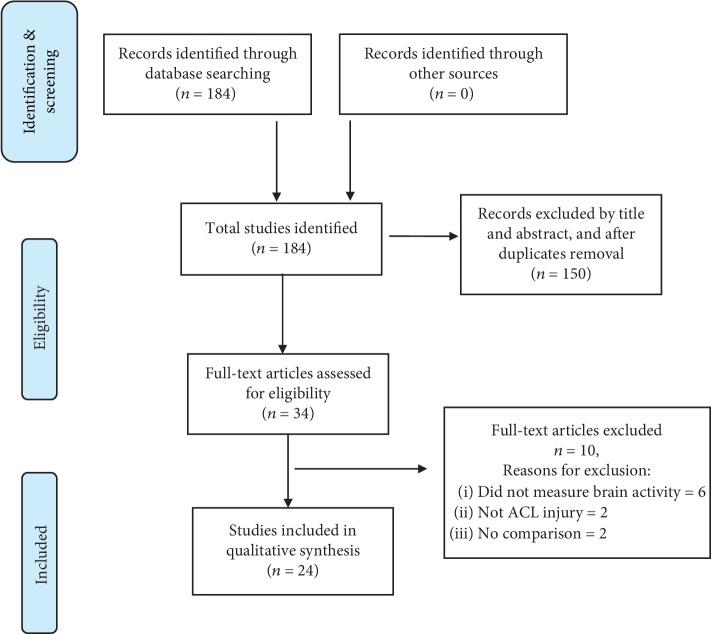
Flow chart of the search strategy and results.

An electronic search was conducted in PubMed, CINAHL, SPORTDiscus, and Cochrane Central Register of Controlled Trials, with no restriction on language or dates of publications, between the months of May 2018 and August 2018. Two independent authors (TN and TS) conducted the search by screening the studies for eligibility after eliminating duplicates. Studies were first selected based on the title and abstract, and only afterwards the inclusion criteria were applied based on full text. The following keywords, and associations between them, were used during the search: brain (or cortical), activation (or activity), neuroplasticity, ACL, and injury (or reconstruction). A detailed syntax of the search can be found in the appendix. Results of the search were organised and presented according to the Preferred Reporting Items for Systematic Reviews and Meta-Analyses (PRISMA) guidelines (Figure 1).

### 2.3. Data Extraction and Presentation

Data extraction of the selected studies was performed by two authors. The following data were extracted from the studies: (a) authors and year of publication; (b) type of study and level of evidence; (c) characteristics of population (e.g., gender, age, and side affected); (d) time since injury/surgery; (e) type of surgery (i.e., for people subjected to ACL reconstruction); (f) presence of other injuries (e.g., menisci, cartilage, or collateral ligaments); (g) physical activity and knee function level; (h) type of rehabilitation performed; (i) methods used to measure brain activity (e.g., EEG, TMS, and fMRI); and (j) task performed during brain activity measurements. In accordance with the Manual of JBI Scoping Reviews, the results of a scoping review should be presented in a diagrammatic or tabular form. This recommendation was followed, and data were presented in tables summarizing information on points (a), (b), (c), (d), (e), (h), (i), and (j). The remaining information was described in the text, in the Results section.

### 2.4. Effect Size

Cohen's *d* was determined as a measure of effect size (ES) of the differences observed [40]. Two types of differences were considered for this analysis: within-subjects (i.e., between limbs of individuals with ACL injury) and between-groups (i.e., between the injured limb of individuals with ACL injury and a matched control limb of healthy participants). Data related to the number of subjects, mean, and standard deviation of the brain activity measurement were used for calculating Cohen's *d*. Effect sizes were classified as small (0.20), medium (0.50), and large (0.80) effects [40].

## 3. Results

A summary of the search strategy is depicted in Figure 1. Initially, 184 articles were identified, and from these, 150 were excluded based on title and abstract screening and removal of duplicates. After assessing the studies for eligibility, a total of 24 studies were included in this review.

Table 1 summarizes the techniques for brain activity measurement and their clinical interpretation. Tables 2 and 3 summarize the main characteristics of the studies using EEG, fMRI, and TMS, respectively, to measure brain activity in participants with ACL injury and healthy controls.

### 3.1. Characteristics of Studies

#### 3.1.1. Population

From the 24 studies, 14 assessed participants with ACL reconstruction (ACLR), six assessed participants with ACL deficiency (ACLD), and two studies included both individuals with ACLR and ACLD. A total of 629 participants with ACL injury were included, 47.3% male and 52.7% female participants—three studies [41–43] did not provide information about the sex of participants. The average (±standard deviation) age of the participants ranged from 24.5 ± 3.7 years (ACLR) to 28.2 ± 4.1 years (ACLD). Three studies [41, 43, 44] did not provide information about the participants' age.

#### 3.1.2. Time since Injury/Surgery and Type of Surgery

The average time since surgery in individuals with ACLR was 30.1 ± 21.9 months, while in participants with ACLD, the average time since injury was 40.8 ± 25.1 months. Five studies did not provide the time since surgery/injury [41–43, 45, 46].

The types of surgery for ACL reconstruction were as follows: (a) graft from the hamstring muscles [29, 45–47]; (b) graft from the patellar tendons [43]; (c) mixed type using grafts from hamstrings and patellar tendons [33, 48, 49]; and (d) mixed type using grafts from hamstrings, patellar tendons, and allografts [35, 50–52]. Four studies [44, 53–55] did not report complete information about the type of graft used for the ACL reconstruction.

#### 3.1.3. Outcomes for Brain Activity

In the selected studies, brain activity was measured by the following: (a) fMRI, during a dynamic task (i.e., cycles of knee flexion-extension) [23, 49]; (b) EEG to perform spectral power analysis during dynamic conditions [29, 47, 56]; (c) EEG to measure somatosensory-evoked potentials (SEPs) as a result of a direct stimulation of the ACL during arthroscopic procedures [41, 45, 46]; and (d) EEG to measure SEPs as a result of common peroneal nerve stimulation in resting conditions [34, 42, 43, 57] (Table 2). The remaining studies used TMS to measure corticospinal excitability (Table 3), mostly during quadriceps contractions (active motor threshold), and only two studies used resting conditions (resting motor threshold) [52, 58]. Other variables measured were as follows: motor-evoked potentials (MEPs) [33, 52, 55, 58, 59], intracortical facilitation (ICF) and short-interval intracortical inhibition (SICI) [52, 55, 59], and long-interval intracortical inhibition (LICI) [59] (please consult Table 1 for more details on the outcomes for brain activity).

#### 3.1.4. Physical Activity Level

Physical activity of the participants with ACL injury at the time of brain activity measurements was assessed by the Tegner scale in 11 studies, ranging from 4.5 (representing a moderately heavy labor) [47] to 7.3 (representing participating in competitive sports) [53]. The Baecke questionnaire was used in one study [23] with an average score of 7.8 ± 1.4; two studies [34, 57] only provided a range of weekly hours (i.e., 1-7 h per week) of physical activity; two studies [33, 52] only provided the preinjury physical activity level; and eight studies [41–43, 45, 46, 56, 58, 59] provided no information about the participants' physical activity level.

#### 3.1.5. Knee Function Level

The International Knee Documentation Committee questionnaire was the most commonly used assessment of knee function. Scores of knee function ranged from 77.2 out of 100 [33] to 88.8 out of 100 [53]. Other measures of knee function included the following: the Lysholm Knee Scoring scale (range = 81.7 ± 13.1 [56] to 86.5 ± 3.9 out of 100 [29]); clinical tests such as the Lachman test, Pivot shift, or the Anterior Drawer tests [42, 43]; and sensation of giving way [34, 57]. The remaining studies [41, 45–47] did not report measures of knee function.

#### 3.1.6. Additional Injuries

A specific description of the ACL injury and the presence of concomitant injuries can be found in 16 studies. Five studies [34, 48, 50, 51, 57] included people with meniscal injuries in addition to the ACL injury; four studies [44, 52, 54, 55] only included people without multiple ligament tears; four studies [29, 42, 43, 47] specifically mentioned to have excluded people with cartilage or meniscal injuries; three studies [53, 56, 59] reported to only have included people with isolated ACL injury.

#### 3.1.7. Rehabilitation

Only 4 out of the 24 studies provide information about rehabilitation performed by people with ACL deficiency or reconstruction. The study of Lepley et al. [33] is the only one to provide a complete description of the rehabilitation program followed by people after ACLR, during 6 months. Other 3 studies reported that participants underwent diversified rehabilitation programs [29, 47, 49].

### 3.2. Effect Size of Differences in Brain Activity

The ES of differences in brain activity between limbs and/or populations could be determined in 15 out of the 24 studies included. Overall, ES ranged from small to large (see Tables 2 and 3).

#### 3.2.1. ACLR vs. Uninvolved

In 8 studies, all using TMS, it was possible to determine the ES of differences in brain activity between the ACLR and the uninvolved limb. The effect size ranged from small (ES = 0.05) [50] to medium (ES = 0.46) [35] with an observed tendency for the ACLR limb to have higher motor thresholds (i.e., lower excitability of the motor cortex) compared to the uninvolved limb (for further details, please see Table 3).

#### 3.2.2. ACLR vs. Healthy

The ES of differences in brain activity between the ACLR limb and a matched healthy limb was determined in 12 studies (8 using TMS, 3 using EEG, and 1 using fMRI). In the TMS studies, the ES of differences in motor threshold ranged from small (ES = 0.16) [59] to large (ES = 1.08) [50], with a tendency for the ACLR individuals to have higher motor thresholds. In EEG studies, ES ranged from medium (ES = 0.3) [45] to large (ES = 1.33) [47], showing higher frontal theta power [47] and abnormalities in SEP reproduction [45, 46]. The study using fMRI showed, in the ACLR limb, higher activation of the contralateral motor cortex, lingual gyrus, and secondary somatosensory area, with a large ES associated to these differences (0.78 to 3.05, Table 2) [49].

#### 3.2.3. ACLD vs. Uninvolved

Only 1 study [53] compared the AMT between limbs of individuals with ACLD. The affected limb showed higher values of AMT with a medium ES (0.27, Table 3).

#### 3.2.4. ACLD vs. Healthy

Two studies [45, 46] measured the SEP mean voltage between individuals with ACLD and healthy participants: one [46] reported no significant differences between groups (ES = 0.21), while the other [45] reported a significantly lower mean SEP voltage in the ACLD group (ES = 1.37). Miao et al. [56] observed a significantly higher band power of all frequencies measured in the ACLD group (ES = 2.43 to 4.07, Table 2).

## 4. Discussion

**Figure 2:**
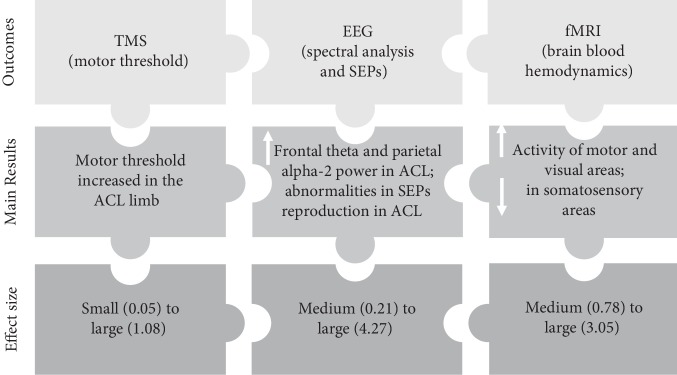
Infographic summarizing the evidence of brain activity changes based on three different measurement techniques in people with ACL injury (legend: ACL: anterior cruciate ligament; EEG: electroencephalography; fMRI: functional magnetic resonance imaging; SEP: somatosensory-evoked potential; TMS: transcranial magnetic stimulation; arrows represent “increase” or “decrease”).

In this scoping review, we included and analysed articles to summarize the current evidence related to the differences in brain activity and corticospinal excitability shown by people with ACL injury. The results from this review (summarized in Figure 2) give us the indication that differences probably exist in brain activity between the ACL-injured and noninjured limbs and/or populations. However, the effect size of these changes seems to differ between the techniques used to measure brain activity. Studies using TMS showed smaller ES in comparison with the EEG studies (and 1 fMRI study). Explanations for this could be related to confounding variables that may have direct impact on the evidence of functional neuroplasticity following ACL injury, as explained hereunder.

### 4.1. Functional Neuroplasticity following ACL Injury

Evidence from the included studies supports the notion of changes both at a sensory and at a motor level after ACL injury. Several studies using different techniques to measure brain activity showed higher levels of activity recorded in the motor areas [23, 29, 47]. Compared to healthy participants, those with ACL injury (with or without reconstruction) recruited the motor cortex to a larger extent [23, 35, 49, 51], even in simple tasks such as joint angle reproduction [29]. A possible explanation for this may be related to the depressed motor cortex excitability shown in people with ACL injury [48, 59], which means that higher motor cortex activity is necessary to achieve efferent drive [60]. Moreover, studies performing spectral power analysis reported higher theta power in the frontal cortex [29, 47]. As this area of the brain has been linked to focused attention and working memory [61], it seems that after an ACL injury, more neurocognitive resources are necessary to conduct simple motor tasks.

Regarding the sensory areas of the cortex, the studies included in this review show evidence of functional neuroplasticity in participants with ACL injury or reconstruction. This may be explained by the disruption in the somatosensory information from the injured ACL [62] which is evidenced by the altered reproduction of SEP following the stimulation of the common peroneal nerve [34, 43] or the remnants of the injured ACL [46]. The lack of proprioceptive input together with nociceptive activity associated to the inflammatory process could be the drivers for a reorganization of the sensory cortex. Studies using fMRI and spectral analysis showed higher levels of activity in certain sensory areas of the brain such as the lingual gyrus, in the visual cortex [23, 29, 47, 49]. This higher need for visual information probably represents a compensatory mechanism due to the deafferentation from the affected knee [63] to allow proper motor control and joint stability [36].

### 4.2. Possible Confounding Variables

Several variables were identified in this review (Tables 2 and 3) that may be considered confounders that influence neuroplastic changes following ACL injury, which will be discussed hereunder.

The outcome measures of brain activity were heterogeneous across the studies (with the exception of both fMRI studies [23, 49] that used similar protocols), which may have contributed to the large range of ES found. EEG techniques used to assess brain activity involved either the measurement of SEPs or spectral power analysis. Within each of these techniques, a variety of protocols was observed. The SEPs were measured either by stimulating the common peroneal nerve [34, 42, 43, 57] or by directly stimulating the injured (or reconstructed) ACL [45, 46]. Regarding spectral analysis, these measurements involved either static conditions (i.e., force reproduction in an isokinetic dynamometer) [47] or dynamic activities (i.e., jogging) [56]. Similarly, different protocols were observed for the TMS measurements which took place in resting conditions [52, 58], or during different intensities of isometric quadriceps contractions [50, 59]. It is possible that these methodological differences partially explain the wide range in effect size for the differences in brain activity between ACL-injured and healthy participants reported in the studies.

One aspect that some studies failed to properly control concerns the characteristics of the population. Variables such as the gender and age of the participants, type of surgery performed in the ACLR patients (Tables 2 and 3), knee function, physical activity, and the presence of pain or other injuries are occasionally missing or incomplete. In other cases, participants were included with very different times from surgery [23, 58]. Although differences in brain activity were generally found after ACL reconstruction, the magnitude of these differences is still unclear as studies have shown different corticomotor excitabilities from 3 months [59] to 46 months after surgery [51].

Another aspect that should be better described in future studies is related to knee function and if the person is classified as *coper* (i.e., resume prior levels of activity without dynamic instability) or *noncoper* [64]. This classification is established after the ACL injury, with or without reconstruction, and can change (e.g., usually from *noncoper* to *coper*) after rehabilitation [64]. As the studies by Courtney et al. [34, 57] demonstrate, *copers* showed changes in SEP cortical representation while *noncopers* did not present such changes. Thus, the question if differences in brain activity dictate a better functional outcome, or if they are related to a full return to sports activities, remains to be answered.

Moreover, very little information was reported about rehabilitation protocols, which is concerning given that several studies [33, 35, 49, 55] explain their results based on their intervention and/or make recommendations about ACL rehabilitation. This lack of information is a significant limitation and highlights the need for future brain neuroplasticity studies to clearly describe rehabilitation protocols conducted before recommendations may be made about rehabilitation.

The lack of information regarding the type of activities practiced on a regular basis and the level of motor skill prior to ACL injury was also another considerable limitation observed in the included studies. Previous research has demonstrated that after six weeks of whole body training [65], or even after 1 h of balance training [66], functional and structural changes in the motor cortex are apparent. In addition, it has been proposed that elite athletes require less brain activation during monopodalic tasks suggesting greater neural efficiency [67]. Thus, it is possible that people with ACL injury who were highly engaged in sports before the injury present differences in brain activity that may be due not only to the injury but also to the neuromotor demands of the sports activities performed before the injury. Moreover, it is unclear to what extent performing sports that promote lower limb asymmetry (e.g., soccer) generates different brain activity patterns. These aspects could be critical for the selection of an appropriate control group and are clearly underreported in the studies described here. Therefore, future reports should comprise a complete profile description including the type, and level, of physical activity performed by the study participants.

Finally, the type of study design may also influence the conclusions about neuroplastic changes after ACL injury. Only one of the included studies followed a longitudinal design, measuring corticomotor excitability over three time points [33]. Longitudinal studies that incorporate measurements before injury and throughout rehabilitation can help in establishing a cause effect between brain neuroplasticity and ACL injury. Very recently, a study was able to use fMRI to measure brain activity before and after ACL injury, observing a weaker functional connection between the left primary sensory cortex (proprioception) and right posterior lobe of the cerebellum (balance and coordination), in the ACL-injured athletes compared to the healthy controls [32]. Hence, this suggests that specific functional brain connectivity patterns may predispose athletes to ACL injury, which may be confirmed with large-scale longitudinal studies that include such brain activity/connectivity measurements in combination with other injury screening tests of healthy athletes.

### 4.3. Future Directions and Recommendations

This scoping review is aimed at providing an in-depth analysis about the evidence of differences in brain activity associated with ACL injury. The discussion about confounding variables and their possible impact on the evidence found leads us to suggest the following recommendations for future studies in this area:

- Participants' demographic information needs to be clearly and completely presented, as well as details regarding the level and type of physical activity performed before the injury and at the time of the brain activity measurements
- Clinical information from participants with ACL injury detailing the injury mechanism, time since injury and surgery, and type of surgery should be thoroughly described
- Detailed information pertaining the rehabilitation protocol of participants with ACL injury should be reported, mainly regarding its duration, frequency, and the type of exercises most frequently performed; the rehabilitation performed by an ACL patient may help to shape the functional neuroplasticity process after the injury but is unfortunately very poorly described in the studies included in this scoping review

Regarding the rehabilitation after ACL injury, it would be relevant if future studies could determine the association between rehabilitation and brain activity in people with ACL injury. Some of the studies included in this review suggest the use of techniques such as TENS [35] and EMG biofeedback [33] to improve cortical excitability. On a different note, a recent review [20] provides a more comprehensive view over the rehabilitation process of ACL patients and proposes a shift towards attentional and environmental aspects of the neuromuscular function, in opposition to a focus in postural alignment and performance of preplanned tasks. Therefore, future longitudinal studies should be aimed at investigating (a) how differences in brain activity evolve throughout time and (b) if rehabilitation is able to influence brain activity and restore it to values similar to healthy matched controls. Only then should it be relevant to do comparative studies to understand which intervention produces the best results.

Another aspect that should be explored, specially by studies using TMS, concerns the corticomotor excitability involving the hamstring muscles. All studies that used TMS to measure motor thresholds and motor-evoked potentials selected the quadriceps (specifically the vastus medialis or lateralis) as the target muscle. Although it is widely accepted that the quadriceps muscle is crucial for proper knee function [68–70], it is surprising that no study measured motor thresholds and MEPs associated with hamstring activation. The hamstring muscles have a direct relationship with knee stability by working in synergy with the ACL in limiting anterior tibial translation [71]. Studies have shown the existence of a ligament muscle arc reflex between the ACL and the hamstrings, revealing the importance of this muscle group during ACL loading [72, 73]. Moreover, recent guidelines for defining a successful outcome after ACL injury or reconstruction clearly show hamstring strength as an important target one and two years after ACL injury and reconstruction [74]. Recent studies have used TMS to elicit motor-evoked potentials in the hamstring muscles of healthy participants in either resting [75] or dynamic [76] conditions. Thus, it seems that the measures taken for the quadriceps musculature might be replicated (i.e., with the necessary methodological adjustments) to the hamstring muscles, providing important information about the neural processes involved in the control of these key muscles for knee joint stability.

Finally, it is currently unknown if, and how, “baseline” brain activity differences are associated with an increased risk of sustaining a future ACL injury. This could be explored by including brain activity measurements in the routine assessment of different sports populations, which would allow to confirm a certain brain activity profile linked to an increased risk of future ACL injury (i.e., similar to the study undertaken by Diekfuss et al. [32]). Consequently, more effective strategies could be implemented to reduce the risk of initial ACL injury.

## 5. Conclusions

This scoping review provides a summary of the current evidence associated to differences in brain activity after an ACL injury and/or reconstruction. Overall, results suggest evidence of functional neuroplasticity following ACL injury (with or without reconstruction), in both sensory (e.g., increased activity in secondary somatosensory area and lingual gyrus) and motor (e.g., lower corticomotor excitability) cortical areas. However, the heterogeneity in the measures of brain activity and corticospinal excitability seems to have an influence on the magnitude of the differences found (i.e., effect size). Moreover, many of the studies failed to control critical variables (e.g., time since injury), which may influence the observed effects. Therefore, it is recommended that future studies in this area should be aimed at minimizing the impact of confounding variables, thus increasing the level of evidence.

## Figures and Tables

**Table 1:** Summary of the techniques used to measure brain activity and clinical interpretations in ACL injury.

Technique		Measurement	Interpretation
EEG	Somatosensory-evoked potentials (SEPs)	Peaks of activity are measured by electroencephalography (EEG) electrodes in the somatosensory cortex after an external stimulus is delivered to the common peroneal nerve or to the ACL (i.e., via arthroscopy)	The ascending stimulus to the somatosensory cortex, following common peroneal nerve stimulation, is detected as P27 component which provides information about the afferent system. Literature shows contradictory information regarding the ability to reproduce SEPs in people with ACL deficiency or reconstruction
	Spectral analysis	EEG signals are measured during a movement (i.e., joint angle or force reproduction). The mean absolute EEG spectral power is divided into different frequencies: delta (0–4 Hz), theta (4.75–6.75 Hz), alpha 1 (7–9.5 Hz), alpha 2 (9.75–12.5 Hz), beta (12.75–18.5 Hz), and gamma (30–80 Hz), corresponding to different levels of activity in different areas of the cortex	It has been suggested that differences in theta power in the frontal cortex may be linked to differences in working memory and focused attention, whereas alpha power is typically inversely related to the neuronal activation. As such, increased alpha power recorded over parietal cortical areas may be interpreted as a deactivation of the somatosensory cortical areas

TMS	Motor-evoked potentials (MEPs)	Muscle response (measured by electromyography), following a transcranial magnetic stimulus (TMS) delivered at the motor cortex travelling down the motor pathways	Decreased MEPs represent less information travelling in the motor pathways to the target muscle
	Motor threshold	Minimum transcranial magnetic stimulation (TMS) intensity necessary to cause a response (MEP) in the target muscle—it is a measure of motor cortex excitability and can be measured at rest (i.e., resting motor threshold), or during an activity (i.e., active motor threshold (AMT))	Motor threshold is inversely related to motor cortex excitability, meaning that people with reduced corticomotor excitability would have a higher motor threshold. A reduction in motor cortex excitability may affect motor output
	Intracortical inhibition (SICI and LICI)	Paired TMS pulses (first, a conditioning subthreshold pulse, followed by a suprathreshold testing pulse) are delivered with varying interstimulus intervals. Short intervals (<5 ms) produce short-interval intracortical inhibition (SICI), while longer intervals (>50 ms) produce long-interval intracortical inhibition (LICI)	SICI is associated with GABAa activity, while LICI is associated with GABAb activity. Higher levels of intracortical inhibition may be associated to lower cortical excitability
	Cortical silent period (CSP)	The cortical silent period (CSP) corresponds to an interruption in voluntary electromyography (time from MEP onset to EMG activity resumption) in the target muscle following TMS. CSP is mediated by GABAb activity at a cortical level	Longer CSP represent higher levels of inhibition, which may lead to muscle inhibition. However, a link between CSP and MEP changes has not been established
	Intracortical facilitation	Similar to intracortical inhibition measurements, paired TMS pulses are used for measuring intracortical facilitation. In this case, a 7 to 30 ms interval between the conditioning and testing pulses is used	Cortical facilitation is mediated by neurotransmitter glutamate onto non-N-methyl-D-aspartate receptors. There is conflicting evidence on whether ICF is changed in people with ACL injury or reconstruction

MRI	Functional MRI during a task	The blood oxygen level-dependent signal is quantified through the blood hemodynamics during a specific task (e.g., knee flexion-extension cycles)	An increased BOLD signal is associated to a higher activity of the respective brain area, which may be associated to reduced efficiency of these cortical regions, in people with ACL injury

**Table 2:** Summary of included EEG and fMRI studies (effect size is presented for between or within-group comparisons).

Study	Level of evidence	Group (*n*, sex, mean age)	Type of surgery; time from injury/surgery	Equipment, outcomes	Task	Results	Effect size, Cohen's *d*
Baumeister et al. [47]	Case-control, 3b	ACLR (*n* = 9, 7M, 2F, age = 25 ± 5) Healthy (*n* = 9, 7M, 2F, age = 24 ± 3)	All hamstrings; 12.0 ± 4.7 months from surgery	EEG, power spectral analysis	Knee extension force reproduction (50% of MVIC)	Significantly higher frontal theta power in ACLR	ACLR vs. healthy, *d* = 0.91-1.33

Ochi et al. [45]	Case-control, 3b	ACLD (*n* = 45, 2M, 24F, age = 29.8) ACLR (*n* = 42, 21M, 21F, age = 32.9) Normal ACL (*n* = 19, 7M, 12F, age = 28.4)	All hamstrings; >13 months after surgery in 38 ACLR participants	EEG—SEP of the ACL	Direct mechanical stimulation of the ACL during arthroscopy (under general anaesthesia)	Mechanically reproduced SEPs were observed in 58% of ACLD, 86% of ACLR, and 100% of healthy ACL No differences in SEP mean voltage between the ACLD (1.3 *μ*V), ACLR (1.27 *μ*V), and normal ACL (1.42 *μ*V)	ACLD vs. ACLR, *d* = 0.06 ACLD vs. healthy, *d* = 0.21 ACLR vs. healthy, *d* = 0.30

Ochi et al. [46]	Case-control, 3b	ACLD (*n* = 32, 16M, 16F, age = 25.5 ± 9.3) ACLR (*n* = 23, 13M, 10F, age = 27.8 ± 10.0) Normal ACL (*n* = 14, 9M, 5F, age = 22.9 ± 12.3)	Hamstring graft in 22 patients and 1 allogeneic fascia lata graft; >18 months after surgery	EEG—SEP of the ACL	Electrical stimulation of the ACL during arthroscopy (under general anaesthesia)	Reproducible SEPs in 47% of ACLD, 96% of ACLR, and 100% of healthy ACL The mean SEP voltage of the ACLD (0.74 *μ*V) was significantly lower (*P* = 0.001) than the healthy group. No differences between ACLD and ACLR	ACLD vs. ACLR, *d* = 0.98 ACLD vs. healthy, *d* = 1.37 ACLR vs. healthy, *d* = 0.65

Miao et al. [56]	Case-control, 3b	ACLD (*n* = 16, all males, age = 26.4 ± 6.3) Healthy (*n* = 15, all males, age = 26.2 ± 3.8)	9 ± 7 months since injury	EEG, power spectral analysis	EEG was recording during the following: (1) Walking (20 m at a natural speed) (2) Jogging (20m) (3) Landing task (25 cm height)	The ACLD group showed a significant increase in band power of all frequencies, during all tasks	ACLD vs. healthy Walking, *d* = 2.07-4.07 Jogging, *d* = 3.58-3.76 Landing, *d* = 2.43-4.46

Valeriani et al. [42]	Case-control, 3b	ACLD (*n* = 19, no information on sex of patients, age = 28.0 ± 4.1) Healthy (*n* = 20, 9M, 11F, age = 23.9 ± 5.2)	Between 12 and 96 months after injury	EEG—SEP of the common peroneal nerve and posterior tibial nerve	Patients relaxed in supine	Seven subjects from the ACLD group showed SEP abnormalities (loss of P27) after common peroneal nerve stimulation	Unable to determine

Valeriana et al. [43].	Case-series, 4	ACLR (*n* = 7, sex and age unknown)	All patellar tendon; time from surgery/injury unknown	EEG—SEP of the common peroneal nerve	Patients relaxed in supine	Absence of cortical P27 response in the injured limb before, and after, ACL reconstruction surgery	Unable to determine

Baumeister et al. [29]	Case-control, 3b	ACLR (*n* = 10, 7M, 3F, age = 27 ± 5) Healthy (*n* = 12, 9M, 3F, age = 25 ± 3)	All hamstrings; 12.5 ± 4.6 months from surgery	EEG, power spectral analysis	Reproduce a given knee angle of 40°	Significantly higher theta and alpha 2 power in ACLR	Unable to determine

Courtney et al. [34]	Case-control, 3b	17 ACLD patients (7M, 10F), divided in the following: *noncopers* (*n* = 4, age = 32), *adapters* (*n* = 10, age = 36), and *copers* (*n* = 3, age = 37)	Overall mean = 68 months after injury: noncopers: 90 months, adapters: 59 months, and copers: 69 months	EEG—SEP of the common peroneal nerve	Patients relaxed in supine	The adapter group showed normal SEPs, 75% of noncopers had normal SEPs, and all copers had altered SEPs	Unable to determine

Courtney et al. [57]	Case-control, 3b	15 ACLD patients (5M, 10F, age = 34), divided in the following: *noncopers* (*n* = 4), *adapters* (*n* = 8), and *copers* (*n* = 3)	Overall mean = 67 months after injury: noncopers: 85 months, adapters: 63 months, and copers: 69 months	EEG—SEP of the common peroneal nerve	Patients relaxed in supine	The adapter group showed normal SEPs, 75% of noncopers had normal SEPs, and all copers had altered SEPs	Unable to determine

Lavender et al. [41]	Case-control, 3b	11 patients: 4 with intact ACL, 6 with complete rupture, and 1 with partial rupture. No information on sex and age	28 months (range = 1-96) after injury	EEG—SEP of the ACL	Electrical stimulation of the ACL during arthroscopy	All intact ACLs (and the partially ruptured) showed reproducible SEPs; ruptured ACL did not show reproducible SEPs	Unable to determine

Kapreli et al. [23]	Case-control, 3b	ACLD (*n* = 17, all male, age = 25.5 ± 5.0) Healthy (*n* = 18, all male, age = 27.0 ± 5.0)	26.2 ± 23.0 months after injury	fMRI	Cycles of 45° knee extension/flexion (1.2 Hz), during 25 s, positioned in supine inside the scanner	ACLD showed less activation of thalamus, PP, PM, cerebellum, iSM1, cSM1, BG GPe, and CMA and showed higher activation of pre-SMA, SIIp, and pITG	Unable to determine

Grooms et al. 2017 [49]	Case-control, 3b	ACLR (*n* = 15, 7M, 8F, age = 21.7 ± 2.7) Healthy (*n* = 15, 7M, 8F, age = 23.2 ± 3.5)	13 hamstrings and 2 patellar tendons; 38.1 ± 27.2 months after surgery	fMRI	4 × 30 s cycles of 45° knee extension/flexion (1.2 Hz), positioned in supine inside the MRI scanner	ACLR showed less activation of iMC and cerebellum and showed higher activation of cMC, lingual gyrus, and iSII	*d* for the following: iMC = 0.78, cerebellum = 3.05, iSM = 2.01, lingual gyrus = 1.14, and cMC = 0.94

ACLR = anterior cruciate ligament reconstruction; ACLD = anterior cruciate ligament deficiency; BG GPe = basal ganglia-external globus pallidus; CMA = cingulated motor area; cMC = contralateral motor cortex; cSM1 = contralateral primary sensorimotor area; EEG = electroencephalography; ES = effect size; F = females; fMRI = functional magnetic resonance imaging; iMC = ipsilateral motor cortex; iSM1 = ipsilateral primary sensorimotor area; iSII = ipsilateral secondary somatosensory area; M = males; pITG = posterior inferior temporal gyrus; PM = premotor cortex; PP = postparietal cortex; pre-SMA = presupplementary motor area; SII = secondary somatosensory area; SEPs = somatosensory-evoked potentials; SIIp = posterior secondary somatosensory area.

**Table 3:** Summary of included TMS studies (effect size is presented for between or within-group comparisons).

Study	Level of evidence	Group (*n*, sex, mean age)	Type of surgery; time from injury/surgery	Outcomes	Task	Results	Effect size, Cohen's *d*
Pietrosimone et al. [35]	Case-control 3b	ACLR (*n* = 28, 9M, 19F, age = 21.3 ± 3.8) Healthy (*n* = 29, 9M, 20F, age = 21.5 ± 2.7)	14 hamstrings, 12 patellar tendons, 2 allografts; 48.1 ± 36.2 months from surgery	AMT	Vastus medialis contraction at 5% MVIC	AMT was significantly higher in the ACLR limb (45.1 ± 15.2) compared to the uninvolved limb (38.4 ± 14.4)—*P* = 0.003 AMT was significantly higher in the ACLR limb (45.1 ± 15.2) compared to healthy controls (37.5 ± 12.7)—*P* = 0.04	ACLR vs. uninvolved limb, *d* = 0.46 ACLR vs. healthy, *d* = 0.54

Pietrosimone et al. [44]	Case series 4	ACLR (*n* = 15, 4 M, 11F, age = unknown)	Unknown; 54.4 ± 12.0 months from surgery	AMT	Vastus medialis contraction at 5% MVIC	The ACLR limb presented average AMT values of 33.2 ± 12.1%T	Unable to determine

Lepley et al. [54]	Case-control 3b	ACLR (*n* = 29, 9M, 20F, age = 21.2 ± 3.7) Healthy (*n* = 29, 9M, 20F, age = 21.5 ± 2.7)	Unknown; 48 ± 36 months from surgery	AMT	Vastus medialis and lateralis contraction at 5% MVIC	The ACLR group showed higher values of AMT (43.9 ± 16.3%2T) compared to healthy controls (37.5 ± 12.7%T), but the significance level of this difference is unknown	ACLR vs. healthy, *d* = 0.43

Lepley et al. [33]	Case-control, 3b	ACLR (*n* = 20, 9M, 11F, age = 20.9 ± 4.4) Healthy (*n* = 20, 9M, 11F, age = 21.7 ± 3.7)	Nine hamstrings, 11 patellar tendons Measurements taken at 3 points: (a) 37.1 ± 15.3 days after injury (b) 15.9 ± 2.4 days after surgery (c) 28.3 ± 2.9 weeks after surgery	AMT, MEP	Vastus medialis contraction at 5% MVIC	Both at presurgery and 2 weeks after surgery, there were no differences between groups in the AMT values At 6 months postsurgery, both the ACLR limb (46.1 ± 8.7%T) and the uninvolved limb (47.4 ± 6.5%T) showed significantly higher AMT compared to the healthy group (36.8 ± 8.6%T) No differences were found for MEP in any time point	For AMT: ACLR vs. uninvolved, *d* = 0.1-0.4 ACLR vs. healthy, *d* = 0.3-1.0

Ward et al. [53]	Case-series, 4	ACLD (*n* = 28, 7M, 21F, age = 22.4 ± 3.7)	Unknown; 52 ± 42 months from injury	AMT	Vastus medialis contraction at 5% MVIC	There were no significant differences between the ACLD limb (46.4 ± 9.9%T) and the uninvolved limb (43.9 ± 8.6%T)—*P* = 0.24	ACLD vs. uninvolved, *d* = 0.27

Kuenze et al. [48]	Case-control, 3b	ACLR (*n* = 22, 12M, 10F, age = 22.5 ± 5.0) Healthy (*n* = 24, 12M, 12F, age = 21.7 ± 3.6)	12 hamstrings, 10 patellar tendons; 37.3 ± 26.3 (hamstring) and 24.5 ± 15.6 (PT) months from surgery	AMT	Vastus medialis contraction at 5% MVIC	The ACLR limb showed a significantly higher AMT (61.8 ± 12.0%T) compared to the uninvolved limb (56.0 ± 14.5%T), but not when compared to the healthy group (63.1 ± 10.3%T)	ACLR vs. uninvolved, *d* = 0.45 ACLR vs. healthy, *d* = 0.17

Luc-Harkey et al. [55]	Case-series, 4	ACLR (*n* = 27, 7M, 20F, age = 21.8 ± 3.2)	18 patellar tendons (remaining unknown); 44.5 ± 36.6 months from surgery	AMT, ICF, SICI, MEP	Vastus medialis contraction at 5% MVIC	No significant differences in AMT were observed between the ACLR (48.2 ± 13.1%T) and the uninvolved limb (46.0 ± 12.6%T). No significant differences were observed for the remaining outcomes	For AMT: ACLR vs. uninvolved, *d* = 0.17

Norte et al. [51]	Case-control, 3b	ACLR (*n* = 72, 32M, 40F, age = 26.0 ± 9.3) Healthy (*n* = 30, 12 M, 18F, age = 22.7 ± 4.6)	34 hamstrings, 29 patellar tendons, 9 allografts; 46.5 ± 58.0 months from surgery	AMT	Vastus medialis contraction at 5% MVIC	AMT values between the ACLR limb (45.2 ± 8.6%T) and the uninvolved limb (44.3 ± 8.4%T) were not significant; however, significant differences were found in the healthy group (39.0 ± 4.1%T)	ACLR vs. uninvolved, *d* = 0.11 ACLR vs. healthy, *d* = 0.81

Norte et al. [50]	Case-control, 3b	ACLR_early_ (*n* = 34, 20M, 14F, age = 22.5 ± 6.3) ACLR_late_ (*n* = 30, 10M, 20F, age = 24.9 ± 5.9) ACLR_OA_ (*n* = 8, 2M, 6F, age = 45.4 ± 7.4) Healthy (*n* = 30, 12M, 18F, age = 22.7 ± 4.6)	29 hamstrings, 26 patellar tendons, 23 allografts; ACLR_early_ = 9.0 ± 4.3, ACLR_late_ = 70.5 ± 41.6, ACLR_OA_ = 115.9 ± 110.0 months from surgery	AMT	Vastus medialis contraction at 5% MVIC	No significant differences in AMT were found between the ACLR limb and the uninvolved limb, both at early (45.8 ± 7.9%T vs. 45.1 ± 7.4%T) and late (42.8 ± 9.1 vs. 42.3 ± 9.5) stages—*P* = 0.60 Both limbs of people with ACLR showed significant differences in AMT compared to the healthy controls (39.0 ± 3.4%T)—*P* < 0.05	All ACLR groups vs. uninvolved limb *d* = 0.05-0.09 All ACLR groups vs. healthy, *d* = 0.6-1.1

Zarzycki et al. [52]	Case-control, 3b	ACLR (*n* = 18, 8M, 10F, age = 21.8 ± 3.3) Healthy (*n* = 18, 8M, 10F, age = 22.2 ± 2.5)	Eight hamstrings, 5 patellar tendons, 3 allografts; 14.0 ± 3.0 days after surgery	ICF, MEP, SICI, and RMT	Participant seated in dynamometer and relaxed	No significant differences were found in RMT between the ACLR limb (61.4 ± 12.4%T) and the uninvolved limb (67.9 ± 15.4%T)—*P* = 0.39 The ACLR limb showed significantly higher RMT compared to healthy controls (55.6 ± 8.2%T)—*P* = 0.001 ACLR group showed higher MEP in both limbs compared to both healthy-matched limbs. ACLR group showed between-limb differences in SICI. No differences were observed in ICF	For RMT: ACLR vs. uninvolved, *d* = 0.46 ACLR vs. healthy, *d* = 0.54

Ward et al. [59]	Case-control, 3b	ACLR (*n* = 18, 12M, 6F, age = 29.6 ± 8.4) Healthy (*n* = 18, 12M, 6F, age = 29.2 ± 6.8)	Unspecified; 69.5 ± 42.5 days after surgery	AMT, CSP, ICF, LICI, MEP, and SICI	Rectus femoris contraction at 10% MVIC	Differences in AMT between the ACLR limb (51.8 ± 9.9%T), the uninvolved limb (50.1 ± 9.2%T), and healthy controls (53.3 ± 8.9%T) were not significant The ACLR limb showed longer CSP compared to the uninvolved limb and healthy controls. No differences were observed for MEP, LICI, and SICI	For AMT: ACLR vs. uninvolved, *d* = 0.17 ACLR vs. healthy, *d* = 0.16

Héroux and Tremblay [58]	Case-control, 3b	ACLD (*n* = 10, 4M, 6F, age = 27 ± 8) Healthy (*n* = 4, 4F, age = 23 ± 3)	22 (range 4-108) months from injury	RMT and MEP	Quadriceps contraction for MEP recordings (details unspecified)	RMT values from the ACLD limb were significantly lower (*P* = 0.02) compared to the uninvolved limb. No comparisons were made to the healthy group No differences were observed for MEP	Unable to determine

%T = percentage of 2.0 tesla; ACLD = anterior cruciate ligament deficiency; ACLR = anterior cruciate ligament reconstruction; AMT = active motor threshold; CSP = cortical silent period; ES = effect size; F = females; ICF = intracortical facilitation; LICI = long-interval intracortical inhibition; M = males; MEP = motor-evoked potential; MVIC = maximal voluntary isometric contraction; RMT = resting motor threshold; SICI = short-interval intracortical inhibition.

## Appendix

### Search Strategy.

The following keywords, and different combinations between them, were used in the electronic search conducted in PubMed, PEDro, CINAHL, SPORTDiscus, and Cochrane Central Register of Controlled Trials databases:

Brain AND Neuroplasticity AND ACL injury

OR

(brain OR cortical) AND neuroplasticity AND ACL (injury OR rupture)

OR

(brain OR cortical) AND (neuroplasticity OR activity) AND ACL (rupture OR deficiency)

OR

(brain OR cortical) AND (neuroplasticity OR activation) AND ACL (rupture OR deficiency)

OR

(brain OR cortical) AND (neuroplasticity OR activation) AND ACL reconstruction.

OR

(brain OR cortical) AND (neuroplasticity OR activation) AND ACL (reconstruction OR repair)

OR

corticomotor AND (neuroplasticity OR excitability) AND ACL (injury OR reconstruction).

OR

(brain OR cortical) AND (neuroplasticity OR activity) AND knee ligament injury.
